# The Evolutionary Origin of Somatic Cells under the Dirty Work Hypothesis

**DOI:** 10.1371/journal.pbio.1001858

**Published:** 2014-05-13

**Authors:** Heather J. Goldsby, David B. Knoester, Charles Ofria, Benjamin Kerr

**Affiliations:** 1 BEACON Center for Evolution in Action, Michigan State University, East Lansing, Michigan, United States of America; 2 Department of Biology, University of Washington, Seattle, Washington, United States of America; 3 Department of Microbiology and Molecular Genetics, Michigan State University, East Lansing, Michigan, United States of America; 4 Department of Computer Science and Engineering, Michigan State University, East Lansing, Michigan, United States of America; University of Lausanne, Switzerland

## Abstract

Reproductive division of labor is a hallmark of multicellular organisms. However, the evolutionary pressures that give rise to delineated germ and somatic cells remain unclear. Here we propose a hypothesis that the mutagenic consequences associated with performing metabolic work favor such differentiation. We present evidence in support of this hypothesis gathered using a computational form of experimental evolution. Our digital organisms begin each experiment as undifferentiated multicellular individuals, and can evolve computational functions that improve their rate of reproduction. When such functions are associated with moderate mutagenic effects, we observe the evolution of reproductive division of labor within our multicellular organisms. Specifically, a fraction of the cells remove themselves from consideration as propagules for multicellular offspring, while simultaneously performing a disproportionately large amount of mutagenic work, and are thus classified as soma. As a consequence, other cells are able to take on the role of germ, remaining quiescent and thus protecting their genetic information. We analyze the lineages of multicellular organisms that successfully differentiate and discover that they display unforeseen evolutionary trajectories: cells first exhibit developmental patterns that concentrate metabolic work into a subset of germ cells (which we call “pseudo-somatic cells”) and later evolve to eliminate the reproductive potential of these cells and thus convert them to actual soma. We also demonstrate that the evolution of somatic cells enables phenotypic strategies that are otherwise not easily accessible to undifferentiated organisms, though expression of these new phenotypic traits typically includes negative side effects such as aging.

## Abstract 2

Experimental evolution of digital organisms suggests that mutagenic side effects associated with performing valuable metabolic work can produce germ-soma differentiation in multicellular organisms.

## Author Summary

Cells within an organism are categorized as “germ” if they are able to grow into a whole new offspring organism or as “soma” if they contribute to the body's functionality but cannot produce an offspring themselves. From an evolutionary perspective, it is important to ask why and how a multicellular organism would demarcate the reproductive potential of its cells. Here we propose the “dirty work hypothesis,” which argues that germ–soma differentiation is an adaptation to allow metabolic work that damages a cell's DNA. Soma can afford to perform this “dirty work,” while germ cells must keep their DNA pristine for future multicellular offspring. We use digital organisms to provide experimental evidence in support of this hypothesis and present an unexpected evolutionary trajectory: multicellular organisms first evolve to confine damaging metabolic work to a subset of cells (which we label “pseudo-soma”) before more complex developmental patterns arise that allow for reproductive division of labor with a proper soma. Finally, we demonstrate that somatic cells allow organisms to evolve valuable functions that are otherwise too damaging to cells; however, they come with the side effect of rapid aging. Similar pressures may have produced reproductive division of labor in other contexts, such as the differentiation of reproductive queens and sterile workers in eusocial insect colonies.

## Introduction

Major transitions in evolution occur when individuals form a higher-level unit that reproduces as a single entity [1]–[3]. Such major transitions can occur when related lower-level units stay together, thus forming a higher-level unit. Examples include the transition from unicellular to multicellular organisms [1]–[3] and the transition from solitary to eusocial insects [1]–[3]. There are several key aspects to such a transition, including the formation of groups (which can be favored by factors such as the ability to avoid predators [4]–[7] or achieve homeostasis [8]) and the specialization of members to take advantage of the benefits of division of labor [1],[2],[9]–[28]. Within these transitions, lower-level units may evolve to exhibit reproductive division of labor (e.g., the germ and somatic cells within a multicellular organism, and the reproductive and worker castes within a eusocial insect colony). Indeed, reproductive division of labor is widely observed throughout nature [1],[2],[9] and has been studied both theoretically [13],[14],[24],[25],[29]–[32] and empirically [33]–[35]. In particular, the existence of non-reproductive somatic cells is a defining feature of multicellular organisms [1],[10],[36],[37].

Several hypotheses have been proposed to explain the evolutionary forces that favor reproductive division of labor within multicellular organisms [8],[36]. Specifically, these hypotheses address the conditions under which it would be adaptive for a multicellular organism to differentiate germ and somatic cells. Bell proposed that germ–soma differentiation evolves as a response to cellular constraints that prohibit simultaneously performing work and reproducing [8],[38]. For instance, in Volvocine algae, an individual cell faces a conflict between motility and cell division because basal bodies cannot both migrate to mitotic poles and stay connected to flagella [35]. Buss proposed that the early sequestration of the germ line results from an evolutionary pressure to suppress mutations acquired through cell division [36]. By identifying germ cells early, the propagule that forms a new multicellular organism has fewer mutations.

Michod and Bendich proposed that reproductive division of labor within complex multicellular organisms may be adaptive, since it ensures the protection of organellar DNA [39]–[43]. Specifically, electron transport in mitochondria and chloroplasts generates reactive oxygen species that, in excess, can lead to oxidative stress which damages DNA [44]. However, a multicellular organism can preserve its genetic material by keeping a subset of cells metabolically quiet (reproductive germ), while other cells perform mutagenic energy metabolism (non-reproductive soma). In this context, reproductive division of labor is a strategy for addressing a trade-off between performing metabolic work that damages the genome (“dirty work”) and protecting the information in the genome. The “dirty work hypothesis” posits that the mutagenic effects associated with metabolism promote the evolution of germ–soma differentiation.

**Figure 1:**
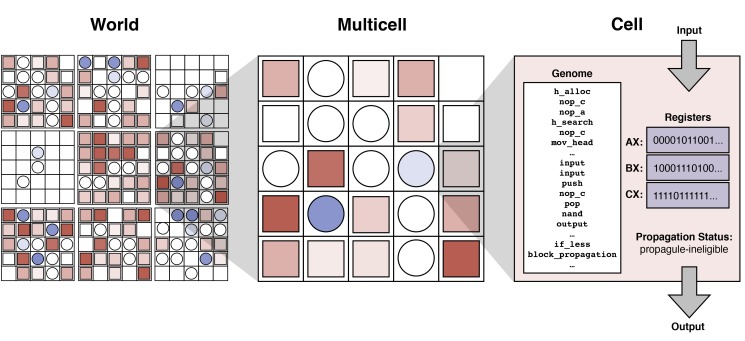
World consisting of digital multicells that each contains up to 25 cells. Cells are either propagule-eligible (circles) or propagule-ineligible (squares). Deeper shades of color represent increased execution of mutagenic functions and, thus, accumulation of mutations in propagule-eligible cells (blue) and propagule-ineligible cells (red). Each cell contains a genetic program that guides its execution; a genome segment encoding one particular computational function (NOT) is shown. The last two depicted lines of the genome demonstrate how simple instructions can be used to evolve phenotypically plastic cells. In particular, the second to last line (*if_less*) specifies that the next instruction (*block_propagation*) should be executed only if the value in the *BX* register (e.g., 10001110100…) is less than the value in the *CX* register (e.g., 11110111111…). In this case, because the value in *BX* is indeed less than the value in *CX*, the *block_propagation* instruction is executed and thus the cell enters the propagule-ineligible state. However, if the values in the registers were different, then the cell could have remained eligible to be used as a propagule.

We tested the dirty work hypothesis using digital evolution [11],[45]–[47], an approach where digital cells are self-replicating computer programs that evolve in an open-ended fashion and are theoretically capable of performing any computational function [46]. For this study, we consider a world of 400 multicellular organisms (“multicells”) that compete for space, where the rate of reproduction of each multicell depends on the number and rate of computational functions being executed by constituent cells (Figure 1). Each cell consists of a program (i.e., its genome), where the instructions in the program allow the cell to self-replicate (producing another cell within the multicell), perform computational functions, access information about its location within the multicell (i.e., its x and y coordinates), and send messages to neighboring cells (see Materials and Methods for details). Each cell has a propagation status that determines whether it can be used as a propagule for a new multicell. By default, a cell is eligible to be a propagule. However, if a cell executes the *block_propagation* instruction, it becomes ineligible to be a propagule (Figure 1). Additionally, new cells within a multicell inherit the propagation status of their parent. Thus, a propagule-eligible cell produces propagule-eligible offspring cells, while a propagule-ineligible cell produces propagule-ineligible offspring cells.

Digital cells perform computational functions (i.e., metabolic work) to acquire resources and enable the multicell to replicate [11]. Each of nine possible logic functions [46] (i.e., NOT, NAND, AND, ORNOT, OR, ANDNOT, NOR, XOR, EQUALS) is associated with a resource that flows into and out of the multicell's environment. To perform these functions, a cell must execute instructions that input numbers, manipulate numbers, and output a result. Mutations within the system replace one instruction with another instruction. For example, within Figure 1, a sample mutation might exchange instruction *input* for instruction *nand*, thus breaking the ability of the cell to perform function NOT. Because functions generally require multiple instructions, multiple mutations must occur for a cell's line of descent to acquire a new function. While mutations typically occur during multicell replication, for each function, we also specify a “function mutagen level” (FML), defined as the per-site probability of mutation each time a function is performed. Function NOT is always non-mutagenic and thus has an FML of 0. For most treatments, the FML of the remaining eight functions is the same. When a cell performs a function, it acquires 5% of the associated resource, and also accumulates any mutagenic effects associated with the function. By performing more types of functions, a cell is able to collect resources more rapidly. However, each performance of one of these mutagenic functions may alter a cell's genetic material, potentially damaging its ability to self-replicate or to collect future resources.

Multicells compete for space within their world in that each time a multicell replicates it replaces an existing multicell. To replicate, a multicell must amass a certain amount of resources (through task performance by its constituent cells). When multicell replication is triggered, a single cell is randomly selected from among the propagule-eligible cells, probabilistically mutated, and then used to seed the new multicell (Figure S1). Any mutations accumulated as the result of propagation are in addition to those accumulated as the result of mutagenic function performance. If a damaged cell is selected as the propagule, then such damage may adversely affect the offspring multicell. If a cell is propagule-ineligible (resulting from either being the offspring cell of a propagule-ineligible cell or having executed the *block_propagation* instruction), then it will not be selected as a propagule. Because each multicell unfolds from a single propagule cell, the cells within a multicell are initially genetically similar, differing only in the mutations accumulated as a result of mutagenic function performance. As such, within this system, it is possible for phenotypic variation to result from either phenotypic noise [35] or phenotypic plasticity, which may result from conditional instruction execution (i.e., genetic regulatory elements [13]) and access to different data (see Materials and Methods for details). In general, phenotypic plasticity is the means by which propagule-ineligible cells arise from propagule-eligible cells.

Our experiments begin with undifferentiated multicells. Specifically, each ancestral multicell is initialized with a single propagule-eligible germ cell that performs a non-mutagenic function (NOT) and then self-replicates, which produces another cell. Over evolutionary time, mutations accrued during the multicell replication process can result in cells that perform different types of functions or behaviors, including blocking their own ability to be selected as a propagule.

The evolution of cells that are both metabolically active and propagule-ineligible (i.e., soma) faces two seemingly insurmountable challenges: First, any cell that performs mutagenic functions will damage its genome, possibly eliminating its ability to perform work or self-replicate. Second, if a cell becomes ineligible to be used as a propagule, it removes itself from the reproductive line of the multicell. For the multicell to thrive, it appears that a subset of cells must perform both damaging actions, while the genetic material that encodes these actions must persist, unexpressed, in other cells (the germ). Certain aspects of our system, including clonal reproduction within well-structured groups, are consistent with circumstances identified by inclusive fitness theory [29],[30] and multilevel selection theory [31],[32], which describe conditions under which reproductive altruism can evolve. However, mutagenic functions increase the genetic differences within multicells, decreasing the relatedness coefficient between cells (from the inclusive fitness perspective) and strengthening selection at the lower level (from the multilevel selection perspective). Noting these impediments, this system can be used to investigate whether reproductive altruism will evolve, and, if so, the sequence of evolutionary steps taken for such germ–soma differentiation.

## Results and Discussion

**Figure 2:**
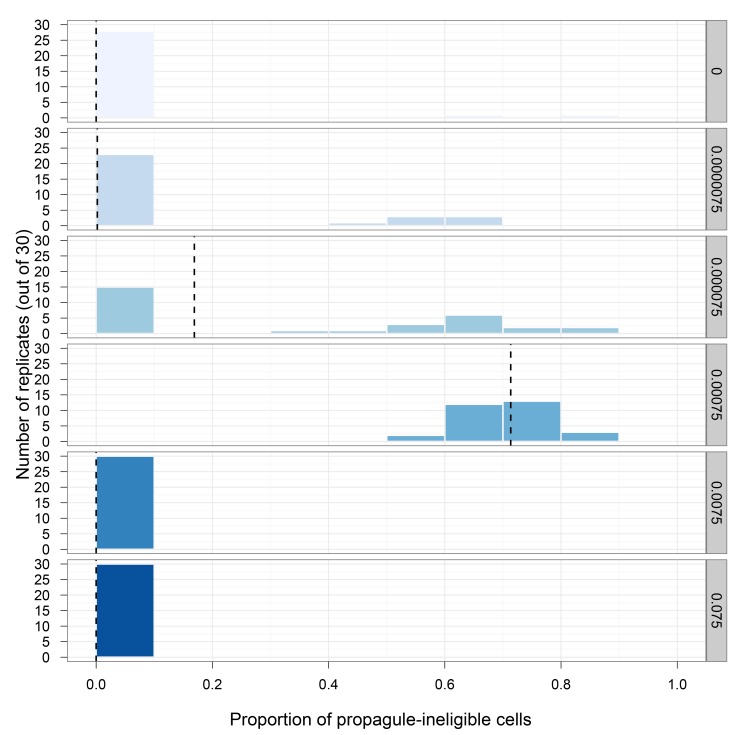
The effect of mutagenic functions on the evolution of somatic cells. Each histogram depicts the results for a specified FML with values ranging from 0.0 to 0.075 per-site probability. Each bar specifies the number of replicates (out of 30) that evolved a given proportion of propagule-ineligible cells. The vertical dashed line is the mean. In the absence of mutagenic effects (FML = 0.0) or at very high levels, propagule-ineligible cells failed to become abundant. At intermediate FMLs, peaking at 0.00075, propagule-ineligible cells evolved. Notably, these propagule-ineligible cells perform a disproportionately large amount of the mutagenic work of the multicell (e.g., propagule-ineligible cells within the 0.000075 and 0.00075 treatments evolved to perform 88.67% and 99.01% of the mutagenic work, respectively). Thus, we consider these propagule-ineligible cells to be soma.

To test the dirty work hypothesis, we performed experiments that varied the FML and examined whether the presence of mutagenic functions favors germ-soma differentiation. For a cell to be classified as soma, it must: (1) be propagule-ineligible and (2) perform a disproportionately large amount of mutagenic work. The results of these evolution experiments are given in Figure 2 and Table S1. With low FMLs, the percentage of propagule-ineligible cells remained low. However, as the FML increased, multicells evolved larger proportions of propagule-ineligible cells (Wilcoxon multiple comparisons rank-sum test, *p*<0.001 for comparisons among treatments with FMLs, varying from 0.0000075 to 0.00075, and the control treatment, where FML was 0.0 [test statistic values specified in Table S2]) until, at the highest mutagen levels (0.0075 and 0.075), the proportion of propagule-ineligible cells decreased.

Next, we examined whether propagule-ineligible cells are true somatic cells that perform disproportionately more mutagenic functions. When propagule-ineligible cells were relatively abundant, we found that they performed a disproportionately large share of functions associated with mutagenic consequences (Table S1), thus we classified them as somatic cells. For FML 0.00075, the propagule workload difference (Materials and Methods), which is the difference between the mean workload of the propagule-ineligible cells and that of propagule-eligible cells, was 63.58 functions, where the average propagule-ineligible cell performed 64.17 mutagenic functions and the average propagule-eligible cell performed 0.59 mutagenic functions (Wilcoxon multiple comparisons rank-sum test, *p*<0.001 for the comparison between the propagule workload difference between FML 0.00075 and the control FML 0.0 [test statistic values specified in Table S3]). Such division of labor was favored because somatic cells, which had removed themselves from consideration as propagule cells, performed the mutagenic functions necessary for the digital multicell to reproduce, while the germ cells maintained the multicell's genetic information in pristine condition.

How did multicells evolve a developmental plan that produced both quiescent germ cells and active somatic cells? From the multicell perspective, there are two potential first steps: (1) evolve additional mutagenic functions (and then later evolve to differentiate propagule-ineligible cells), or (2) differentiate propagule-ineligible cells (and then later have this subset of cells take on additional mutagenic functions). The first approach has some immediate benefits for the reproductive rate of the multicell but at the cost of putting the genetic information of the multicell in peril. The second approach has no initial benefit and thus this process relies on genetic drift to produce differentiation.

**Figure 3:**
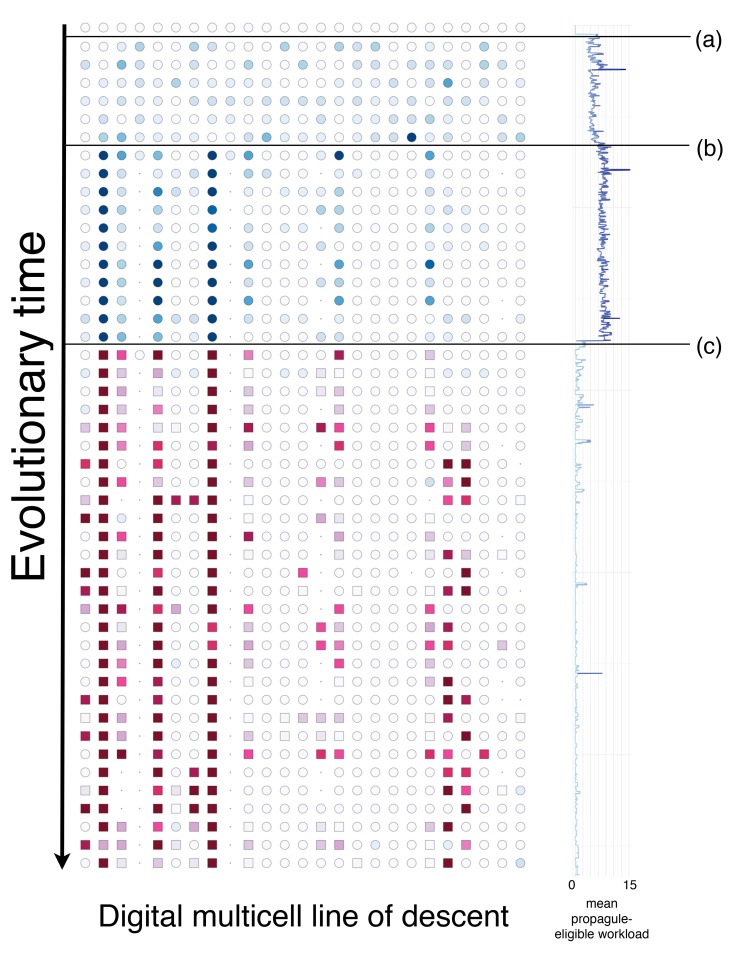
The evolutionary trajectory of an example digital multicell. Each row represents a digital multicell along a study lineage. Circles signify propagule-eligible germ cells. Squares signify propagule-ineligible cells. Color intensity represents the number of mutagenic functions performed by propagule-eligible cells (blue) and propagule-ineligible cells (red). Within this lineage, the ancestral multicell began with all the cells in a pristine state. After (**a**), the propagule-eligible cells evolved to perform a variety of mutagenic functions and then to segregate their workload, thus producing pseudo-somatic cells (**b**). Eventually, these pseudo-somatic cells became ineligible to be used as propagules. At this point, they became true somatic cells (**c**). After this point, the somatic cells continued to perform mutagenic functions at a high level, while the germ cells remained quiescent. The mean propagule-eligible workload is correlated with the number of mutations that would be passed on to an offspring digital multicell. Notably, when germ–soma differentiation occurred (**c**), the mean propagule-eligible workload decreased, thus protecting the multicell's propagule from mutational damage.

To understand the route taken by the multicells that evolved germ–soma differentiation, we examined the line of descent from each replicate evolved with FML 0.00075. For each multicell along these lineages, we recorded the behavior of each constituent cell. Analyzing these lineages, we found that the multicells followed an unanticipated variation on route 1. Figure 3 depicts one such lineage. The multicell first split the workload heterogeneously among the propagule-eligible cells, where some propagule-eligible cells (which we call “pseudo-soma”) performed many more mutagenic functions than others. This innovation protected the genetic material in some of the propagule-eligible cells while the pseudo-soma dramatically increased the multicell's replication rate. Next, the multicell evolved to make these pseudo-somatic cells into actual somatic cells by blocking their ability to be selected as a propagule and thus guaranteeing that only high-fidelity germ cells were used to produce the next generation of offspring multicells.

In all replicates, the multicell made use of phenotypic plasticity to divide the workload heterogeneously prior to the advent of somatic cells. To assess the mechanisms employed by cells within various replicates, we performed instruction-level knock out experiments. Specifically, to analyze a multicell, we modified a copy of its propagule's genome, replacing all instances of the coordination instruction of interest with a neutral instruction. We then seeded this modified propagule into a test environment and observed how the change affected the speed at which a digital multicell acquired resources. If the multicell's performance was degraded as compared with the unmodified version, it meant that the coordination instruction type we knocked out was required for optimal performance. We found that in 29 of 30 replicates with FML 0.00075, cells evolved conditional execution of instructions based on communicated signals (indicated by losing functionality when the *retrieve_message* instruction was knocked out), while in 12 of 30 replicates, cells evolved conditional execution of instructions based on location information (indicated by losing functionality when the *get_xy* instruction was knocked out). Analyses also revealed that lineages from different replicates made use of both communication and location information over the course of evolutionary time. In particular, in some replicates, lineages made use of both types of mechanisms, whereas other lineages alternated between mechanisms, and yet other lineages only ever made use of one. Germ–soma differentiation evolved upon a foundation of these existing mechanisms for phenotypic plasticity which originally split the workload among propagule-eligible cells. Text S1 describes the final genome from two different lineages in detail.

To provide evidence that the evolutionary trajectory shown in Figure 3 was common among replicates, we identified the point of reproductive differentiation, where the percentage of propagule-ineligible cells rose sharply in each study lineage. We then examined the distribution of the workload across propagule eligible and ineligible cells (Table S4). Specifically, we compared the mean workload of propagule-eligible (germ) cells before and after the evolution of propagule-ineligible cells. In every replicate, the workload of the germ cells decreased. These data are consistent with an evolutionary pathway that involves creating pseudo-soma and then blocking propagation for these pseudo-somatic cells (leaving all the actual germ cells with a low workload). In Text S2, we describe a mathematical model that further supports a wide range of conditions where the existence of pseudo-soma would be advantageous.

To further assess whether this pathway was favored by evolution, we performed an additional control at FML 0.00075 in which we eliminated the ability of digital multicells to evolve pseudo-somatic cells. To do so, we redistributed the mutagenic effects associated with performing a function to equally degrade all propagule-eligible cells. Specifically, each time a propagule-eligible cell performed a function, the mutagenic consequences associated with that function were applied to the propagule-eligible cell that had incurred the smallest mutagenic workload. Thus, the mutagenic workload of all propagule-eligible cells remained relatively balanced throughout the lifetime of the multicell. Under these conditions, substantially fewer propagule-ineligible cells evolved (proportion of propagule-ineligible cells: 6.4±2.3 [mean ± standard error] vs. 71.6±1.3 in the original treatment; Wilcoxon rank-sum (W), *p*<0.001, W = 899) and those that did appear were substantially less effective (6.3±3.1 mean propagule workload difference versus 63.6±2.0 in the original treatment; Wilcoxon rank-sum, *p*<0.001, W = 870).

The evolution of true soma sets the stage for a kind of defection at the level of digital cells. Specifically, suppose a mutation removes the *block_propagation* instruction in one cell within a developing multicell. This cell (and all its cellular offspring) become eligible to be picked as propagules, despite the mutagenic functions they perform. Thus, in the competition to be selected as the propagule within a single multicell, such a mutant has an intrinsic advantage over a cell with an intact *block_propagatio*n instruction. However, when this mutant propagule founds a new multicell, the potential for its propagules to carry additional (destructive) mutations is greater than a multicell founded by propagule encoding the development of true soma (and thus maintaining pristine germ cells). As a result, the mutant gains its advantage within a genetically heterogeneous multicell at the expense of compromising the functionality of offspring of a genetically homogeneous multicell. From a multilevel selection perspective, selection within multicells favors the mutant, while selection between multicells disfavors the mutant. Several models for the evolution of multicellularity have focused on analogous conflict between defectors and cooperators within a multicell [2],[40]–[43],[48]. Aspects of our multicell life cycle (Figure S1), including the single cell bottleneck and relatively few cell divisions in multicell development, weaken selection within multicells and consequently hinder the evolution of the mutant. (We note that these same factors increase cellular relatedness within multicells, favoring the evolution of cellular altruism from an inclusive fitness perspective [29],[30].) To explore the possibility of defection, we focused on populations that evolved true soma (under FML = 0.00075) and converted a fraction of the cells within each multicell in the world to defectors (by knocking out the *block_propagation* instruction). Even though the defecting mutants rise in frequency initially, they eventually become extinct (Figure S4). Thus, despite the short-term advantage within multicells, defection is selected against in our system over the longer term. Interestingly, if the incidence of mutagenic functions in the germ decreases further following the evolution of soma, the *generation* of defecting propagules would become less likely [49].

To understand whether the evolution of somatic cells opened up phenotypic niches that were previously inaccessible to undifferentiated multicells, we created an extreme environment in which functions were associated with successively higher mutagenic consequence, a “ramped” FML (Materials and Methods; Table S6). We then compared the evolution of functions in this environment with a control treatment, where we did not allow cells to remove themselves from consideration as propagules. When propagule-ineligible cells were allowed, 27/30 replicates evolved soma, and 24/27 evolved to perform the most damaging logic function, which was associated with an FML of 0.006. For the control treatment in which propagule-ineligible cells were not allowed, only 3/30 replicates evolved to perform the most damaging logic function, and they did so at a substantially reduced level (236.2±22.0 function performances in the control vs. 1109.5±38.3 with propagule-ineligible cells allowed, Wilcoxon rank-sum, *p*<0.001). These data support the hypothesis that somatic cells open up previously inaccessible phenotypic niches. Although we observe that some functional specialization (i.e., workload heterogeneity among propagule-eligible cells) precedes reproductive specialization (i.e., germ-soma differentiation), the existence of reproductive specialization supports further functional specialization, which is in line with current hypotheses regarding the evolution of division of labor [12].

**Figure 4:**
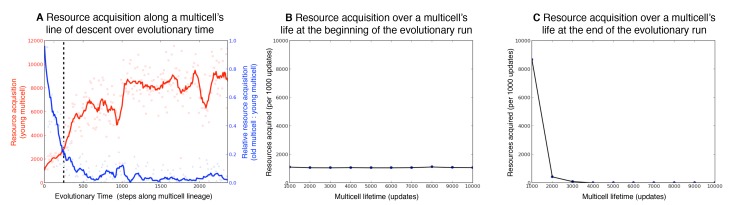
Measurements of digital multicell aging over evolutionary time. Over evolutionary time (**A**), a digital multicell evolves to consume resources more rapidly when young (resources consumed 0–1,000 updates of its lifetime; red line; circles) and relatively much more slowly when old (ratio of resources consumed late within its lifetime [9,000–10,000 updates], as compared with early in its lifetime [0–1,000 updates], blue line; triangles). The black dashed vertical line is the time point at which propagule-ineligible cells evolved. (**B**) At the beginning of the evolutionary run (the first step along the line of descent), the resource acquisition rate of a digital multicell remains constant throughout its lifetime (0–10,000 updates). (**C**) At the end of the evolutionary run (the final step along the line of descent), the resource acquisition rate of a digital multicell sharply decreases throughout its lifetime (0–10,000 updates). These results demonstrate antagonistic pleiotropy between resource consumption early in life and aging later in life.

Within organic systems, the mutagenic effect of oxidative stress causes organisms to age [50]–[52]. Likewise, we also observe that our digital multicells age: their ability to perform functions and acquire resources degrades as a result of performing mutagenic functions. For all treatments with mutagenic functions, ancestral multicells existed in an immortal state in which metabolic efficiency never degraded. However, the evolution of mutagenic functions corrupted their immortality by damaging their genetic program over the lifetime of the multicell. These results are an example of the classic antagonistic pleiotropy explanation for aging [53]. Specifically, the multicells embrace mutagenic functions that are advantageous when the multicell is young but lead to a more rapid deterioration of functionality. Notably, multicells that produce somatic cells age faster than undifferentiated multicells. We compared the rate of resource acquisition for a multicell when it was young (0–1,000 updates) to when it was old (9,000–10,000 updates) (Figure 4). For example, an older multicell with a relative resource acquisition rate of one consumes the same amount of resources as it did in its youth. The multicells that evolved to include soma exhibited a median relative resource acquisition rate of 0.182 late in life, compared with multicells that could not evolve soma, which exhibited a relative resource acquisition rate of 0.395 (Wilcoxon rank-sum, *p*<0.001, W = 748). These data suggest that somatic cells enable the performance of more toxic functions and thus more rapid aging.

Previous studies have suggested that tradeoffs play a role in the evolution of reproductive division of labor [3],[10],[13],[14]. Here we explore a tradeoff between protecting genetic information and performing mutagenic (“dirty”) work. When the dirty work is relatively “clean” (FML<0.000075), we observe that the selective pressure for reproductive division of labor is minimized and thus germ–soma differentiation does not occur. At the opposite extreme, when FMLs are too high (>0.00075), embracing the performance of mutagenic functions is too damaging for cells. Essentially, any execution of these functions destroys the genome of the cell, including its replication and functional abilities, such that performance of these functions is selected against. As a result, multicells are unable to take the first steps toward reproductive division of labor. However, at intermediate FMLs, propagule-eligible cells can take these first steps by executing some mutagenic work; and eventually the tradeoff is circumvented by the differentiation of somatic cells to execute the lion's share of the dirty work and germ cells to preserve a clean genome.

While our experiments demonstrate that the tradeoff between preserving genetic information and conducting dirty work is a sufficient condition to promote the evolution of reproductive division of labor, it is not the only condition under which such differentiation will be favored. Indeed, in some cases, multiple conditions that select for the evolution of division of labor may occur simultaneously. In particular, Buss's hypothesis that early germline sequestration is favored because it limits the number of cell divisions and thus mutation accumulation [36] is compatible with the dirty work hypothesis. In the same vein, there is a connection between our result and findings from two-locus population genetic models [40]–[43]. In these models, alleles at one (modifier) locus control the number of cell divisions in the germline or the mutation rate in the germline. A modifier allele that sequesters germ earlier (i.e., fewer germ divisions) or lowers the germ mutation rate (i.e., fewer mutations per germ division) has the result of reducing the opportunity to pass destructive mutations (at the second locus) to offspring. Even if such sequestration is costly, modifiers can still evolve when the risk of destructive mutations is sufficiently high. In our system, multicells also evolve to minimize mutational damage to the transmitted genome. Instead of reducing the number of divisions, however, the germ cells are protected by abstinence from mutagenic work (effectively lowering their mutation rate). The critical metabolism is shifted to the somatic cells, where the accumulated mutations cannot pass to offspring multicells.

Within eukaryotic organisms, reactive oxygen species in mitochondria and chloroplasts can corrupt DNA, which sets the stage for the evolution of reproductive differentiation if multicellularity has already evolved [39]–[43]. However, similar evolutionary trajectories may occur at scales above and below the level of the organism. Specifically, within colonies of eusocial insects, queens and workers can exhibit two orders of magnitude difference in life span [54]–[56], which may be caused by the effects of oxidative stress [55],[56]. In particular, queens may be producing fewer reactive oxygen species or may be more resistant to oxidative stress [55],. There is evidence that flight behavior (associated with foraging) raises metabolic requirements and thus reactive oxygen species production in bees [57]. By refraining from foraging, the queen may be allocating mutagenic activities to workers while maintaining the colony's information for the next generation. The same information-work tradeoff may also have motivated a switch from RNA to DNA as the molecule of heredity. According to the RNA world hypothesis [58], RNA initially served as both a carrier of genetic material and a catalyst for metabolic work. However, RNA instability may have motivated a shift to DNA genomes and catalytic proteins [59]. This is a type of molecular division of labor, ensuring both high fidelity transmission of hereditary information and the execution of critical chemical work.

## Materials and Methods

For these experiments, we used the digital evolution framework within the Evolutionary Algorithms Library (EALib; https://github.com/dknoester/ealib). This framework is based on the Avida digital evolution system [46]. The specific software used for all experiments and analyses conducted here can be accessed in the “gls” branches of https://github.com/heathergoldsby/ealib (for the evolutionary algorithm library) and https://github.com/heathergoldsby/ealife (for experiment-specific configurations). Our data are available in the Dryad Digital Repository [60]. Each trial consists of a world with 400 digital multicells, which are all seeded at the beginning of every trial. Each multicell starts with a single digital cell, but can grow to contain up to 25 cells arrayed in a 5×5 toroidal grid. For each treatment, we run 30 replicates to account for the stochastic nature of the evolutionary process.

Each digital cell has a genome, which is a circular list of instructions, and a virtual CPU that it uses to execute its instructions. The ancestor digital cell used to start the experiments has a genome that encodes self-replication and the performance of a simple logic function (NOT). The set of instructions that can be mutated into the genome include the standard Avida instructions [46], which enable basic computational operations and operations that change the order in which instructions in the genome are executed. Additionally, we have provided communication instructions (e.g., send message, receive message), location-sensing instructions [11], and an instruction to block propagation (Table 1).

**Table 1:** Avida instructions used to coordinate behavior and differentiate.

Instruction	Description
*get_xy*	Place the cell's x and y coordinates in registers BX and CX, respectively
*send_message*	Send a message containing the contents of registers BX and CX to the neighbor the cell is facing
*broadcast_message*	Broadcast a message containing the contents of registers BX and CX to all neighboring cells
*retrieve_message*	Retrieve the contents of a message and place them in registers BX and CX
*block_propagation*	Change a cell from propagule-eligible to propagule-ineligible
*if_propagule_eligible*	Execute the subsequent instruction if the cell is propagule-eligible
*if_propagule_ineligible*	Execute the subsequent instruction if the cell is propagule-ineligible
*mov_head*	Move the instruction pointer head, which determines the instruction that is executed next, to a different position in the genome
*if_label*	Execute the subsequent instruction if the complement of the label was just copied as part of cell replication
*if_less*	Execute the next instruction if the value in register BX is less than the value in register CX

Within this study, cells have access via mutation to a variety of instructions that enable them to self-replicate, perform functions, coordinate their behavior, and set their propagation status. In this table, we describe several of the instructions that, when executed, enable a cell to coordinate its behavior with other cells (i.e., *get_xy, send_message, retrieve_message, broadcast_message*), remove itself from consideration as a propagule (i.e., *block_propagation*), and conditionally execute instructions within its genome (i.e., *if_propagule_eligible, if_propagule_ineligible, if_label, if_less, mov_head*).

Digital multicells compete for space within their world. To replicate, they must consume 500 units of resource. A digital multicell acquires resources when its digital cells perform functions, which are a form of metabolic work. There are nine possible Boolean logic functions within these experiments (i.e., NOT, NAND, AND, ORNOT, OR, ANDNOT, NOR, XOR, EQUALS) [45],[46]. Each of these logic functions is associated with a resource, which flows into and out of the environment like nutrients in a chemostat. In each update (the standard unit of time, where each digital cell executes 30 instructions), for each resource, one unit of resource flows in and 1% of the available resource flows out to limit total resource accumulation. When a digital cell performs a logic function, it acquires 5% of the associated resource. When a digital multicell replicates, one of its propagule-eligible cells is randomly selected, potentially mutated, and then used as the propagule for the offspring digital multicell, which displaces another randomly chosen multicell.

For each function, we also define an FML, which is the probability that each site within the genome will be mutated to another instruction after the function is executed. For all experiments, the function NOT is non-mutagenic (i.e., FML_NOT_ = 0.0). For treatments in the standard environment, the FML values for all functions besides NOT are the same. However, for the ramped FML treatments, we associate each type of function with an increasingly mutagenic effect by multiplying the base FML of function NAND by increasing integer values. Table S4 provides an example of the FMLs for standard and ramped treatments.

We define *propagule workload difference* as the mean workload of propagule-ineligible cells minus the mean workload of propagule-eligible cells. A cell's workload is defined as:
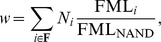
where 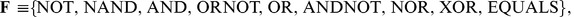 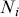 is the number of times function 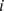 is performed, 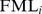 is the mutagenic level of function 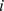, and 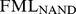 is the base FML (i.e., the mutagenic level of function NAND). For standard treatments, 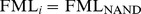 for all 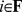 and thus the workload is a measure of the number of mutagenic functions performed.

## Supporting Information

Figure S1 **Life cycle of a multicell.** A multicell begins as a single propagule cell (**A**). This propagule cell self-replicates to produce additional cells that may perform mutagenic work in order to acquire resources. Some cells may also become propagule-ineligible as a result of executing the *block_propagation* instruction, leading to a potentially differentiated multicell (**B**). When the multicell has amassed enough resources, one of the propagule-eligible cells is randomly selected (**C**) and is used to seed a new multicell that displaces one of the other multicells present within the world.
(TIF)

Figure S2 **Ancestor and evolved genomes.** Within this figure, we present three genomes. (**A**) The ancestor genome used for all cells within the initial population. (**B**) An evolved genome of cells within a multicell that differentiated based on location information (i.e., the x and y coordinates of the cells). (**C**) An evolved genome of cells within a multicell that differentiated based on communication among the cells, as well as location information. All three genomes contain instructions required for a cell to self-replicate (i.e., the cell offspring allocation and cell replication loop instructions; highlighted in green). Additionally, both evolved genomes contain a section of instructions that produce propagule-ineligible cells (i.e., the *block_propagation* conditional instructions; highlighted as blue transitioning to red). Both evolved genomes also contain a “soma loop,” where propagule-ineligible cells continue to loop over the same sequence of instructions performing large amounts of mutagenic work (highlighted in red). Although we highlight blocks of instructions that contribute to specific functionality, it must be emphasized that each instruction mutates independently. Thus, any coordination, work, or phenotypic plasticity strategy is evolved in pieces over evolutionary time.
(TIF)

Figure S3
**The optimal fraction of the multicell to allocate to pseudo-soma.** The pseudo-soma fraction is a function of the probability of destructive mutations in the pseudo-soma (*μ*) and the ratio of pseudo-soma to quiescent germ baseline resources (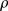). Here we have set 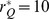 and 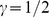.(TIF)

Figure S4 **Defectors within digital multicells.** Within our experiments, it is possible for “defectors” to arise. Defectors can be seen as cells within the digital multicell that prioritize their own fitness considerations over those of the multicell. In the context of a differentiated multicell, one kind of defector is a cell that never executes the *block_propagation* instruction and is always eligible to be used as a propagule. We performed further analyses to understand the fate of such defectors in our experimental system. Specifically, we used the populations evolved under FML = 0.00075. After the final evolutionary time point, we introduced a percentage of defectors into an evolved population. We created defectors by randomly selecting cells, knocking out the *block_propagation* instruction from the cell's genomes, and indicating these cells were eligible to be used as propagules. We then ran the population for an additional 2,000 updates. Here, we depict the proportion of defectors over time. Given their higher chance of being picked as a propagule within the initial mixed multicells, the defectors rise in frequency in the short term. However, because multicells founded by a defector propagule are more likely to pass accumulated mutations to their offspring multicells, they are at a long-term disadvantage. Eventually the defectors go extinct in our system.
(TIF)

Table S1 **Proportion propagule-ineligible cells and propagule workload difference values.** We calculate the mean propagule workload difference by taking the mean workload of the propagule-ineligible cells (see Materials and Methods) and subtracting the mean workload of the propagule-eligible germ cells. For treatments that evolved substantial proportions of propagule-ineligible cells, the mean propagule workload difference is strongly positive, indicating that the propagule-ineligible cells are performing the vast majority of the mutagenic functions. However, when the proportion of propagule-ineligible cells is small (i.e., treatment 0.0000075), the mean propagule workload difference is negative. These rare propagule-ineligible cells likely result from recent mutations that introduce the *block_propagation* instruction to the genome. These values suggest that, when first introduced, the *block_propagation* instruction may tend to disrupt other functions, causing the propagule-ineligible cells to perform less work than the propagule-eligible (germ) cells.
(DOC)

Table S2 **Statistics for comparisons among proportion of propagule-ineligible cells.** Treatments vary the FML. For these comparisons, we used the Wilcoxon multiple comparisons rank-sum test with Holm adjustment method. Here we report the test statistic (W).
(DOCX)

Table S3 **Statistics for comparisons among propagule workload difference values.** Treatments vary the FML. For these comparisons, we used the Wilcoxon multiple comparisons rank-sum test with Holm adjustment method. Here we report the test statistic (W).
(DOCX)

Table S4 **Decrease in mean propagule-eligible workload after the evolution of propagule-ineligible cells.** For each replicate in the 0.00075 FML treatment, we report the mean propagule-eligible (germ) cell workload before and after the identified point at which the multicell transitions to include propagule-ineligible cells. Specifically, data are first smoothed (sliding window of length 100) and the pre- and post-points are 100 steps along the line of descent in either direction. These data indicate that for all 30 replicates the mean propagule-eligible workload decreases after the evolution of propagule-ineligible cells.
(DOC)

Table S5 **Density-dependent resource acquisition.** The proportion of propagule-ineligible cells evolved under varying resource conditions with an FML of 0.00075. In general, for our experiments, each function was associated with a pool of limited resources such that each time a cell performed the function, it consumed a percentage of the available resource (results are described by the “limited resources all functions” row). When these resources become “unlimited” (i.e., a cell receives the same amount of reward regardless of the number of times the function has been performed), then the cells do not evolve to perform mutagenic functions; nor do they evolve substantial amounts of propagule-ineligible cells. We illustrate this by associating function NOT with an unlimited amount of resources, while all other functions are associated with limited resources. In this case, multicells do not evolve to perform any additional mutagenic functions or propagule-ineligible cells.
(DOC)

Table S6 **Sample function mutagen levels.** For each of the nine logic functions that were rewarded within this study, we provide sample function mutagen levels for a standard treatment and a ramped treatment. Notably, in the ramped treatment, the mutagenic consequences associated with performing some of the functions (e.g., EQUALS) are substantially larger than the mutagenic consequences associated with performing other functions (e.g., NAND).
(DOC)

Text S1 **Phenotypic plasticity case studies.** We present the final genome from two different lineages in detail. Specifically, we explain how the instructions within each genome allow for phenotypically plastic behavior.
(DOCX)

Text S2 **Mathematical model for pseudo-somatic cells.** We describe a mathematical model that further supports a wide range of conditions where the existence of pseudo-soma would be advantageous.
(DOCX)

## Abbreviations

FML: Function Mutagen Level
