# Supplementary figures and images for: The Evolutionary Origin of Somatic Cells under the Dirty Work Hypothesis
Source: PLoS Biol. 2014 May 13;12(5):e1001858. doi: 10.1371/journal.pbio.1001858 (PMC4019463; doi:10.1371/journal.pbio.1001858)

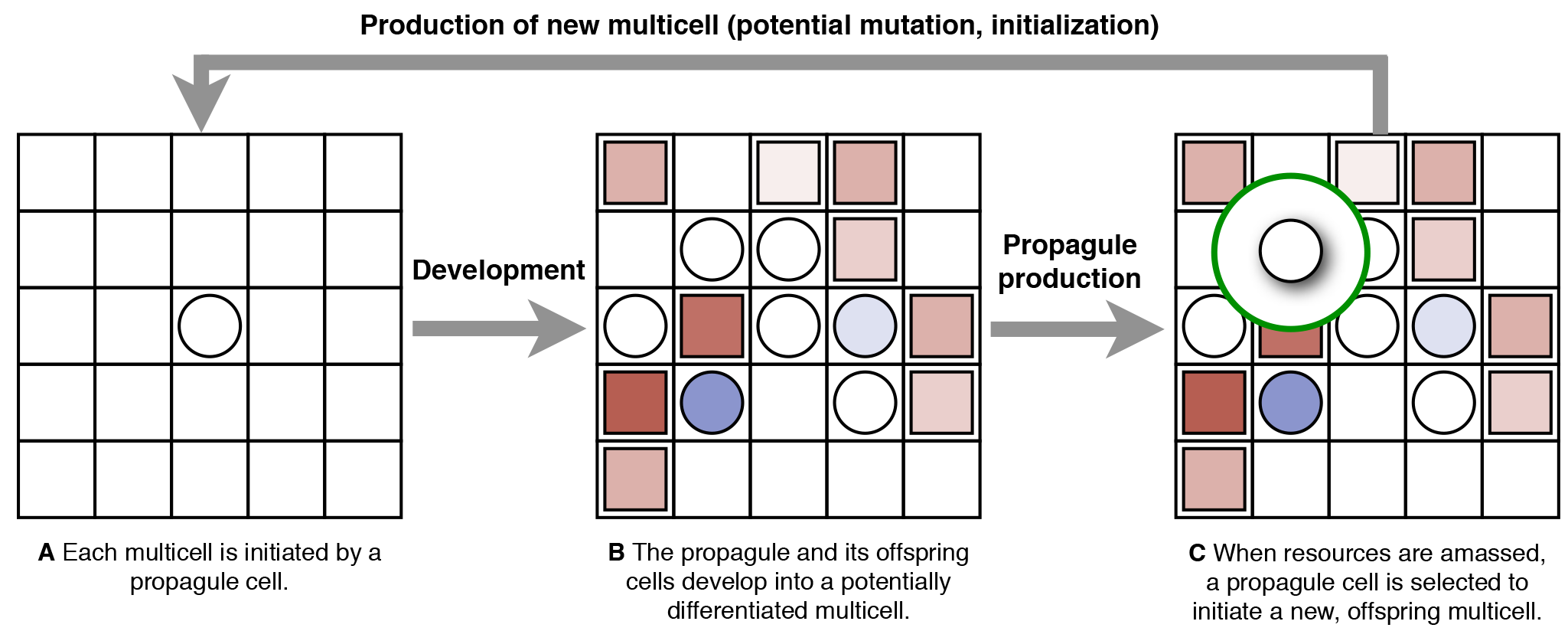

Supplement: Figure S1 — Life cycle of a multicell. A multicell begins as a single propagule cell (A). This propagule cell self-replicates to produce additional cells that may perform mutagenic work in order to acquire resources. Some cells may also become propagule-ineligible as a result of executing the block_propagation instruction, leading to a potentially differentiated multicell (B). When the multicell has amassed enough resources, one of the propagule-eligible cells is randomly selected (C) and is used to seed a new multicell that displaces one of the other multicells present within the world. (TIF) [file pbio.1001858.s001.tif]

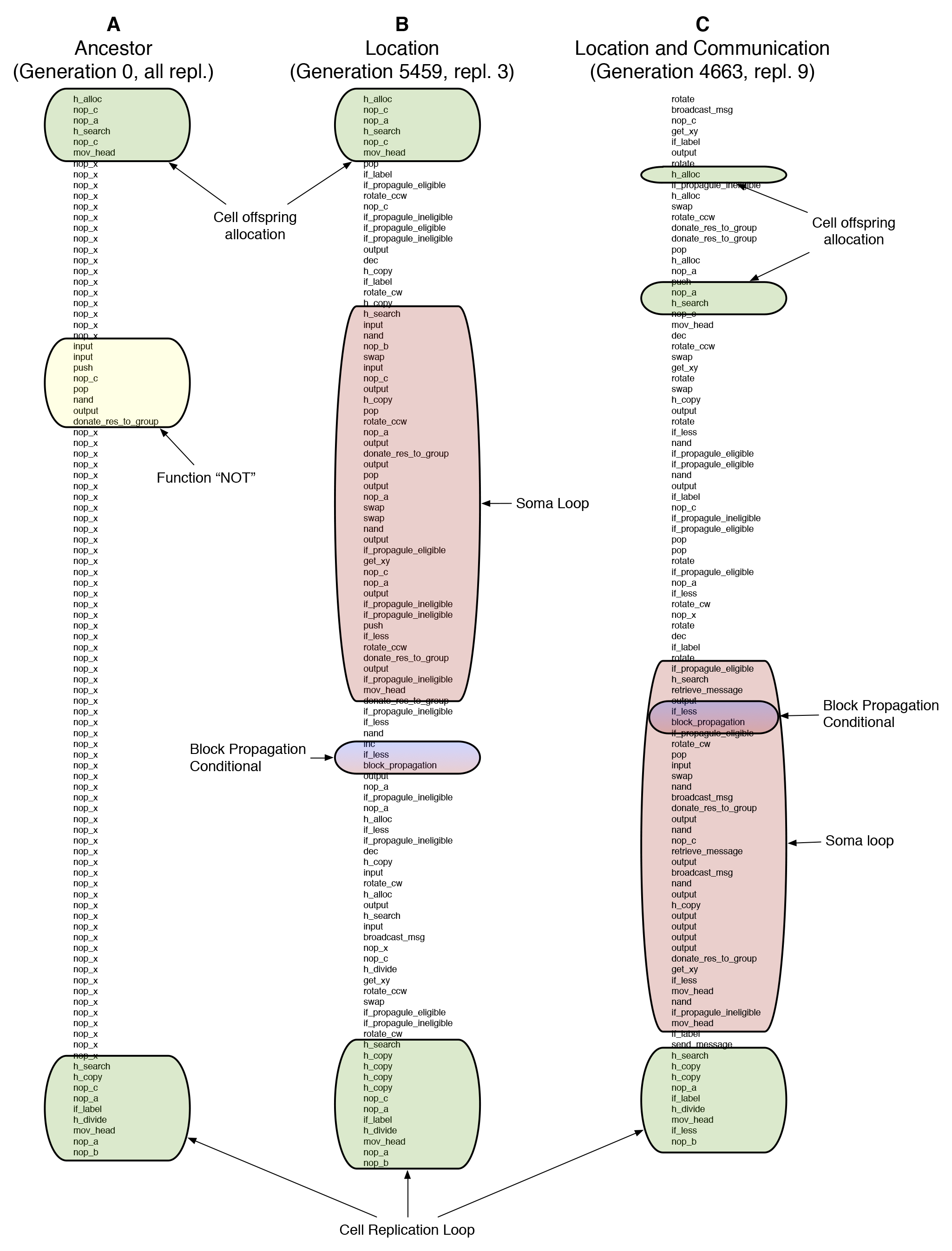

Supplement: Figure S2 — Ancestor and evolved genomes. Within this figure, we present three genomes. (A) The ancestor genome used for all cells within the initial population. (B) An evolved genome of cells within a multicell that differentiated based on location information (i.e., the x and y coordinates of the cells). (C) An evolved genome of cells within a multicell that differentiated based on communication among the cells, as well as location information. All three genomes contain instructions required for a cell to self-replicate (i.e., the cell offspring allocation and cell replication loop instructions; highlighted in green). Additionally, both evolved genomes contain a section of instructions that produce propagule-ineligible cells (i.e., the block_propagation conditional instructions; highlighted as blue transitioning to red). Both evolved genomes also contain a “soma loop,” where propagule-ineligible cells continue to loop over the same sequence of instructions performing large amounts of mutagenic work (highlighted in red). Although we highlight blocks of instructions that contribute to specific functionality, it must be emphasized that each instruction mutates independently. Thus, any coordination, work, or phenotypic plasticity strategy is evolved in pieces over evolutionary time. (TIF) [file pbio.1001858.s002.tif]

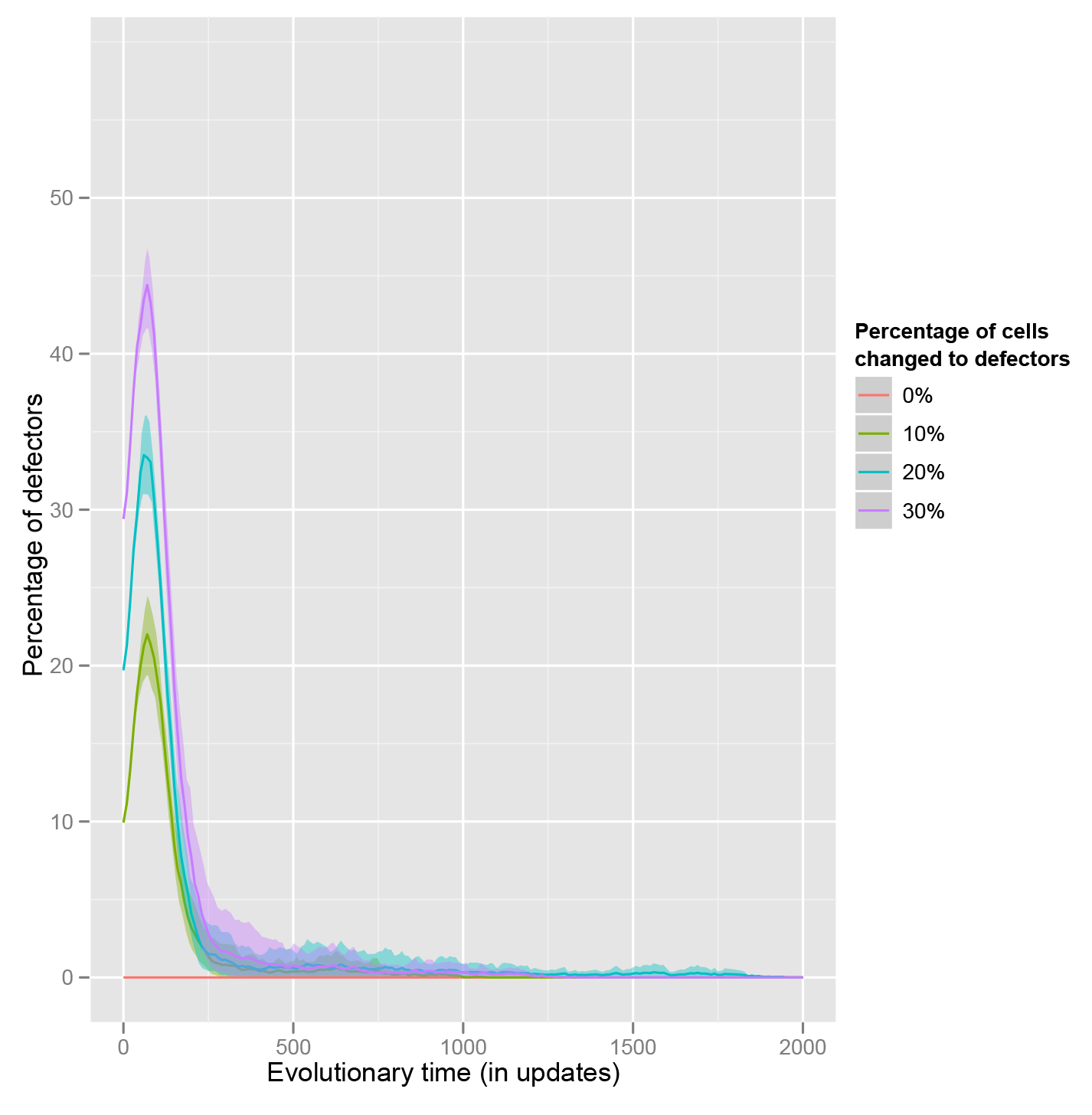

Supplement: Figure S4 — Defectors within digital multicells. Within our experiments, it is possible for “defectors” to arise. Defectors can be seen as cells within the digital multicell that prioritize their own fitness considerations over those of the multicell. In the context of a differentiated multicell, one kind of defector is a cell that never executes the block_propagation instruction and is always eligible to be used as a propagule. We performed further analyses to understand the fate of such defectors in our experimental system. Specifically, we used the populations evolved under FML = 0.00075. After the final evolutionary time point, we introduced a percentage of defectors into an evolved population. We created defectors by randomly selecting cells, knocking out the block_propagation instruction from the cell's genomes, and indicating these cells were eligible to be used as propagules. We then ran the population for an additional 2,000 updates. Here, we depict the proportion of defectors over time. Given their higher chance of being picked as a propagule within the initial mixed multicells, the defectors rise in frequency in the short term. However, because multicells founded by a defector propagule are more likely to pass accumulated mutations to their offspring multicells, they are at a long-term disadvantage. Eventually the defectors go extinct in our system. (TIF) [file pbio.1001858.s004.tif]
