# Supplementary material for: The Evolutionary Origin of Somatic Cells under the Dirty Work Hypothesis
Source: PLoS Biol. 2014 May 13;12(5):e1001858. doi: 10.1371/journal.pbio.1001858 (PMC4019463; doi:10.1371/journal.pbio.1001858)
Supplement: Table S1 — Proportion propagule-ineligible cells and propagule workload difference values. We calculate the mean propagule workload difference by taking the mean workload of the propagule-ineligible cells (see Materials and Methods) and subtracting the mean workload of the propagule-eligible germ cells. For treatments that evolved substantial proportions of propagule-ineligible cells, the mean propagule workload difference is strongly positive, indicating that the propagule-ineligible cells are performing the vast majority of the mutagenic functions. However, when the proportion of propagule-ineligible cells is small (i.e., treatment 0.0000075), the mean propagule workload difference is negative. These rare propagule-ineligible cells likely result from recent mutations that introduce the block_propagation instruction to the genome. These values suggest that, when first introduced, the block_propagation instruction may tend to disrupt other functions, causing the propagule-ineligible cells to perform less work than the propagule-eligible (germ) cells. (DOC) [file pbio.1001858.s005.doc]

| **Function Mutagen Level** | **Mean Proportion Propagule-Ineligible Cells ± SE** | **Mean Propagule Workload Difference ± SE** |
| --- | --- | --- |
| 0.0 | 0.050 ± 3.46 | 0 ± 0 |
| 0.0000075 | 0.132 ± 4.43 | -26.01 ± 13.33 |
| 0.000075 | 0.320 ± 5.97 | 32.05 ± 13.48 |
| 0.00075 | 0.716 ± 1.33 | 63.58 ± 1.96 |
| 0.0075 | 0.005 ± 0.31 | 0.14 ± 0.01 |
| 0.075 | 0.004 ± 0.22 | 0.00 ± 0.00 |
