# Supplementary material for: The Evolutionary Origin of Somatic Cells under the Dirty Work Hypothesis
Source: PLoS Biol. 2014 May 13;12(5):e1001858. doi: 10.1371/journal.pbio.1001858 (PMC4019463; doi:10.1371/journal.pbio.1001858)
Supplement: Table S2 — Statistics for comparisons among proportion of propagule-ineligible cells. Treatments vary the FML. For these comparisons, we used the Wilcoxon multiple comparisons rank-sum test with Holm adjustment method. Here we report the test statistic (W). (DOCX) [file pbio.1001858.s006.docx]

|  | 0 | 0.0000075 | 0.000075 | 0.00075 | 0.0075 |
| --- | --- | --- | --- | --- | --- |
| 0.0000075 | 860 |  |  |  |  |
| 0.000075 | 869 | 529 |  |  |  |
| 0.00075 | 866 | 529 | 435 |  |  |
| 0.0075 | 866 | 608 | 547 | 550 |  |
| 0.075 | 862 | 473 | 413 | 411 | 317 |
