# Supplementary material for: The Evolutionary Origin of Somatic Cells under the Dirty Work Hypothesis
Source: PLoS Biol. 2014 May 13;12(5):e1001858. doi: 10.1371/journal.pbio.1001858 (PMC4019463; doi:10.1371/journal.pbio.1001858)
Supplement: Table S3 — Statistics for comparisons among propagule workload difference values. Treatments vary the FML. For these comparisons, we used the Wilcoxon multiple comparisons rank-sum test with Holm adjustment method. Here we report the test statistic (W). (DOCX) [file pbio.1001858.s007.docx]

|  | 0 | 0.0000075 | 0.000075 | 0.00075 | 0.0075 |
| --- | --- | --- | --- | --- | --- |
| 0.0000075 | 750 |  |  |  |  |
| 0.000075 | 870 | 346 |  |  |  |
| 0.00075 | 900 | 299 | 344 |  |  |
| 0.0075 | 900 | 240 | 154 | 162 |  |
| 0.075 | 900 | 182 | 60 | 20 | 98 |
