# Supplementary material for: The Evolutionary Origin of Somatic Cells under the Dirty Work Hypothesis
Source: PLoS Biol. 2014 May 13;12(5):e1001858. doi: 10.1371/journal.pbio.1001858 (PMC4019463; doi:10.1371/journal.pbio.1001858)
Supplement: Table S4 — Decrease in mean propagule-eligible workload after the evolution of propagule-ineligible cells. For each replicate in the 0.00075 FML treatment, we report the mean propagule-eligible (germ) cell workload before and after the identified point at which the multicell transitions to include propagule-ineligible cells. Specifically, data are first smoothed (sliding window of length 100) and the pre- and post-points are 100 steps along the line of descent in either direction. These data indicate that for all 30 replicates the mean propagule-eligible workload decreases after the evolution of propagule-ineligible cells. (DOC) [file pbio.1001858.s008.doc]

| **Replicate** | **Pre-Transition Propagule-Eligible (Germ) Cell Workload** | **Post-Transition Propagule-Eligible (Germ) Cell Workload** | **Propagule-Eligible (Germ) Cell Workload Difference (Post-Pre)** |
| --- | --- | --- | --- |
| 1 | 2.18 | 0.57 | -1.61 |
| 2 | 4.94 | 0.71 | -4.23 |
| 3 | 7.84 | 0.88 | -6.96 |
| 4 | 5.86 | 1.30 | -4.56 |
| 5 | 5.94 | 0.91 | -5.03 |
| 6 | 4.86 | 0.35 | -4.51 |
| 7 | 4.79 | 0.10 | -4.69 |
| 8 | 6.07 | 2.94 | -3.12 |
| 9 | 7.77 | 1.86 | -5.90 |
| 10 | 2.71 | 0.72 | -1.98 |
| 11 | 6.00 | 0.86 | -5.15 |
| 12 | 6.10 | 2.88 | -3.22 |
| 13 | 5.83 | 0.94 | -4.90 |
| 14 | 7.18 | 1.66 | -5.52 |
| 15 | 6.43 | 1.52 | -4.91 |
| 16 | 6.47 | 0.91 | -5.56 |
| 17 | 6.36 | 1.94 | -4.42 |
| 18 | 1.67 | 0.30 | -1.36 |
| 19 | 6.36 | 1.84 | -4.53 |
| 20 | 5.10 | 0.63 | -4.47 |
| 21 | 3.96 | 0.25 | -3.72 |
| 22 | 5.89 | 1.68 | -4.21 |
| 23 | 6.73 | 1.69 | -5.04 |
| 24 | 6.49 | 1.24 | -5.26 |
| 25 | 4.34 | 0.69 | -3.65 |
| 26 | 5.65 | 0.37 | -5.28 |
| 27 | 6.02 | 0.96 | -5.06 |
| 28 | 7.18 | 1.46 | -5.72 |
| 29 | 6.12 | 1.21 | -4.91 |
| 30 | 5.41 | 2.01 | -3.40 |
|  |  |  |  |
| **Mean** |  |  | -4.43 |
|  |  |  |  |
|  |  |  |  |
|  |  |  |  |
|  |  |  |  |
