# Supplementary material for: The Evolutionary Origin of Somatic Cells under the Dirty Work Hypothesis
Source: PLoS Biol. 2014 May 13;12(5):e1001858. doi: 10.1371/journal.pbio.1001858 (PMC4019463; doi:10.1371/journal.pbio.1001858)
Supplement: Table S5 — Density-dependent resource acquisition. The proportion of propagule-ineligible cells evolved under varying resource conditions with an FML of 0.00075. In general, for our experiments, each function was associated with a pool of limited resources such that each time a cell performed the function, it consumed a percentage of the available resource (results are described by the “limited resources all functions” row). When these resources become “unlimited” (i.e., a cell receives the same amount of reward regardless of the number of times the function has been performed), then the cells do not evolve to perform mutagenic functions; nor do they evolve substantial amounts of propagule-ineligible cells. We illustrate this by associating function NOT with an unlimited amount of resources, while all other functions are associated with limited resources. In this case, multicells do not evolve to perform any additional mutagenic functions or propagule-ineligible cells. (DOC) [file pbio.1001858.s009.doc]

| **Treatment** | **Proportion of propagule-ineligible cells** |
| --- | --- |
| Limited resources all functions | 0.716 ± 1.33 |
| Unlimited resources for non-mutagenic function; limited resources for other functions | 0.045 ± 0.031 |
| Unlimited resources for all functions | 0.122 ± 0.050 |
