# Supplementary material for: The Evolutionary Origin of Somatic Cells under the Dirty Work Hypothesis
Source: PLoS Biol. 2014 May 13;12(5):e1001858. doi: 10.1371/journal.pbio.1001858 (PMC4019463; doi:10.1371/journal.pbio.1001858)
Supplement: Table S6 — Sample function mutagen levels. For each of the nine logic functions that were rewarded within this study, we provide sample function mutagen levels for a standard treatment and a ramped treatment. Notably, in the ramped treatment, the mutagenic consequences associated with performing some of the functions (e.g., EQUALS) are substantially larger than the mutagenic consequences associated with performing other functions (e.g., NAND). (DOC) [file pbio.1001858.s010.doc]

| **Function Name** | **Standard FML** | **Ramped FML** |
| --- | --- | --- |
| NOT | 0.0 | 0.0 |
| NAND | 0.00075 | 0.00075 x 1 = 0.00075 |
| AND | 0.00075 | 0.00075 x 2 = 0.00150 |
| ORNOT | 0.00075 | 0.00075 x 3 = 0.00225 |
| OR | 0.00075 | 0.00075 x 4 = 0.003 |
| ANDNOT | 0.00075 | 0.00075 x 5 = 0.00375 |
| NOR | 0.00075 | 0.00075 x 6 = 0.0045 |
| XOR | 0.00075 | 0.00075 x 7 = 0.00525 |
| EQUALS | 0.00075 | 0.00075 x 8 = 0.006 |
