# Supplementary material for: The Evolutionary Origin of Somatic Cells under the Dirty Work Hypothesis
Source: PLoS Biol. 2014 May 13;12(5):e1001858. doi: 10.1371/journal.pbio.1001858 (PMC4019463; doi:10.1371/journal.pbio.1001858)
Supplement: Text S1 — Phenotypic plasticity case studies. We present the final genome from two different lineages in detail. Specifically, we explain how the instructions within each genome allow for phenotypically plastic behavior. (DOCX) [file pbio.1001858.s011.docx]

**Supporting Information Text S1: Phenotypic Plasticity Case Studies.**

Supporting Information Figure S2 depicts three different genomes from the FML=0.00075 treatment. The first genome (Figure S2A) is the ancestral genome used to seed each multicell at the beginning of an evolutionary run. This genome comprises three different functional blocks separated by a series of *nop_x* instructions (*nop_x* is a “no operation” instruction has no effect other than to consume a single virtual CPU cycle; it is a placeholder that provides evolution with a "blank tape"). The first block of instructions, shown highlighted in green, allocates additional memory into which instructions for the cell's offspring may be copied. The second block, highlighted in yellow, performs the NOT logic function and donates any acquired resources to the multicell. Finally, the third block, again highlighted in green, copies the parent cell's genome into the memory allocated for the cell's offspring.

The second and third genomes in the figure (Figures S2B and S2C) are the final genomes along unrelated lines of descent from replicates 3 and 9, respectively. Genome B, which is 5459 generations removed from the ancestor, uses a strategy that is based solely upon its location in the multicell to fully differentiate into either germ or soma. Genome C, at generation 4663, uses a strategy that relies upon location and communication via messaging to differentiate.

While the specific strategies and instructions used by the two evolved genomes differ, they are logically similar. For example, both genomes contain blocks of instructions that perform cell offspring allocation and cell replication. Moreover, both genomes contain two blocks of instructions that are critical for germ-soma differentiation. The first, highlighted in blue-red, is the block propagation conditional. These instructions test for a genome-specific condition, and if true, block propagation of the cell (one of the conditions for a cell being considered soma). For example, genome B blocks propagation when the y-component of its x-y location in the multicell is non-zero, while genome C blocks propagation when it receives a message whose data is numerically less than an internally calculated value. Each genome also contains a block of instructions we call the "soma loop," which is a block of instructions that performs mutagenic logical functions (shown highlighted in red). Cells that have blocked their propagation execute these instructions in an infinite loop. That is, once a cell has elected to become a soma, it can never leave the soma loop.

The combination of these two strategies, a condition for blocking propagation and an infinite soma loop, has significant ramifications for the multicell. Specifically, cells that are locked in their soma loop are no longer able to replicate. Thus, while soma may be accumulating mutations as a result of their workload, these mutants are not, in fact, competing for space with germ cells--they are simply contributing resources to the multicell. Moreover, the condition under which a cell chooses to block its propagation implicitly requires a multicell with a large cell population. For example, genome B, a location-based strategy, will not block propagation until cells have replicated beyond the first row of the multicell. This condition is stronger in the case of genome C, which requires neighboring cells to communicate with each other prior to propagation being blocked.
