# Supplementary material for: The Evolutionary Origin of Somatic Cells under the Dirty Work Hypothesis
Source: PLoS Biol. 2014 May 13;12(5):e1001858. doi: 10.1371/journal.pbio.1001858 (PMC4019463; doi:10.1371/journal.pbio.1001858)
Supplement: Text S2 — Mathematical model for pseudo-somatic cells. We describe a mathematical model that further supports a wide range of conditions where the existence of pseudo-soma would be advantageous. (DOCX) [file pbio.1001858.s012.docx]

**Supporting Information Text S2: Mathematical model for pseudo-somatic cells.**

Here we consider a multicell in which all cells are germ, but they differ in function performance. For simplicity, we shall assume that every cell falls into one of two states: an active “pseudo-soma” state or a relatively quiescent “true-germ” state. Ignoring development (growth and differentiation of cells), we consider the multicell starting with $n$ cells. Suppose that a fraction$f$of these cells are pseudo-soma and $(1-f)$ are true germ cells. Each time step, we imagine that every cell performs functions (very few for the true germ and many for the pseudo-soma). The functions performed by pseudo-soma are mutagenic and destroy the performing cell with some probability. Thus, the number of functional pseudo-soma cells can decrease over time. Let $P_{t}$ and $Q_{t}$ be variables to refer to the number of functional pseudo-soma cells and quiescent germ cells at time $t$, respectively. Assume that each functional pseudo-soma cell and quiescent germ cell contributes $\rho_{P}P_{t}^{\gamma-1}$ and $\rho_{Q}Q_{t}^{\gamma-1}$ resources to the multicell at time step $t$. Here $\rho_{P}$ and $\rho_{Q}$ give the amount of resources contributed by a single isolated pseudo-soma cell and single isolated germ cell, respectively. We assume $\rho_{P}> \rho_{Q}$, that is, more resources are gathered by pseudo-soma cells than germ cells. The parameter $\gamma$controls the concavity of the functional relationship between resource acquisition rate per cell and the number of cells. We assume $0< \gamma<1$, that is, resource acquisition per cell decreases with the number of cells performing functions. Such density dependent resource acquisition is necessary for evolving digital multicells that perform a diverse suite of functions (see Table S5 for further details). The accumulated resources in the multicell at time $T$is:

$R_{T}=\sum_{t=0}^{T-1} (\rho_{P}P_{t}^{\gamma}+\rho_{Q}Q_{t}^{\gamma}).$ [S1]

Because a quiescent cell is immortal, $Q_{t}=n(1-f)$ for all $t$. The number of functional pseudo-soma cells $(P_{t})$ is expected to decrease over time. For simplicity, we will assume that a fraction $\mu$ will experience a destructive mutation. Thus, $P_{t}=nf(1-\mu)^{t}$.

In order to replicate, suppose that the multicell requires $R^{*}$ resources. Then, by equation [S1], the $T$ value that solves the following equation gives the time of multicell replication:

$R^{*}=\rho_{P}n^{\gamma}f^{\gamma}\left\{\sum_{t=0}^{T-1} (1-\mu)^{\gamma t}\right\}+ \rho_{Q}n^{\gamma}(1-f)^{\gamma}T.$ [S2]

Letting $\rho=\rho_{P}/\rho_{Q}$ and $r_{Q}^{*}=R^{*}/(n^{\gamma}\rho_{Q})$ and simplifying equation [S2] yields:

$r_{Q}^{*}=\rho f^{\gamma}\frac{1-(1-\mu)^{\gamma T}}{1-(1-\mu)^{\gamma}}+(1-f)^{\gamma}T.$ [S3]

Let $T^{*}$ be the value of $T$that solves equation [S3]. Although there is no analytical solution for .., one can solve for its value numerically. For simplicity, we shall assume that after a multicell attempts reproduction, the parent is “reset” to its native state with all pseudo-soma cells functional. The reproductive attempt is successful if a quiescent germ cell or a functional pseudo-soma cell is chosen as the propagule. Given that we know the time of multicell reproduction $(T^{*})$, the rate of growth of the population of multicells can be calculated as:

$\lambda=\{2-f(1-(1-\mu)^{T^{*}})\}^{\frac{1}{T^{*}}}.$ [S4]

We let $f_{\text{opt}}=\mathrm{argmax}_{f}(\lambda)$. In Supporting Information Figure S3, we show $f_{\text{opt}}$ as a function of $\mu$ and $\rho$. As pseudo-soma functions become less mutagenic ($\mu$ decreases) or as the resource acquisition rate of pseudo-soma cells relative to true germ cells increases ($\rho$ increases), the optimal fraction of pseudo-soma increases. We note that a large portion of parameter space has $0<f_{\text{opt}}<1$; thus, a type of division of labor is optimal in this simple model.
